# T97. CANNABIS USE IMPACTS SYMPTOM PRESENTATION IN ANTIPSYCHOTIC NAIVE PATIENTS IN FIRST EPISODE OF PSYCHOSIS (FEP)

**DOI:** 10.1093/schbul/sby016.373

**Published:** 2018-04-01

**Authors:** Luccas Coutinho, Cinthia Higuchi, Daniel Azevedo Cavalcante, Rodrigo Bressan, Quirino Cordeiro, Cristiano Noto, Ary Gadelha

**Affiliations:** 1 Universidade Federal de São Paulo; 2 Faculdade de Ciências Médicas da Santa Casa de São Paulo

## Abstract

**Background:**

Psychotic disorders induced by cannabis may present distinct symptomatic profile, course and underlying biology. Most studies on symptomatic effects of cannabis exposure are limited by examining patients after antipsychotic treatment. We investigated if antipsychotic naive FEP patients that reported cannabis use present higher symptom’s severity and if affects deferentially any of standard psychosis dimensions (positive, negative, disorganized, excitement and depressive).

**Methods:**

The sample comprised 194 antipsychotic naive FEP individuals. The baseline assessment was performed right after the admission at the emergency room and the follow-up assessment two months after antipsychotic treatment. The cannabis exposure was measured by ASI-6 (Addiction Severity Index) and additional questions addressing the relation to onset of psychotic disorders and how many times cannabis has been used. Cannabis use was reported by 41,2% of patients, and 25.8% reported heavy use (more than 50 times).

Dimensional psychopathology was assessed with the Positive and Negative Syndrome Scale (PANSS) and the symptom dimensions were constructed based on previous studies (positive, negative, disorganized, depressive, excitement). Considering cannabis use, we analyzed the following variables: 1 - Number of days of cannabis consumption in the last 30 days (Acute Use); 2 – Age of first cannabis use; 3 – Total cannabis use lifetime, categorized in no use, less than 50, more than 50 (total use).

**Results:**

The mean age was 25.52 (sd=7.17), mean duration of untreated psychosis was 176 days (sd= 291) and most of the subjects were male (63%). Acute use of cannabis was associated with higher scores in the positive symptom dimension (p =0.017, df= 40, R-squared = 0.132). Also, the earlier age of first cannabis use was related to higher presentation scores of the negative symptom dimension (p = 0.002, df = 33, R-squared = 0.238). No significant association was found between any cannabis exposure variable and other symptomatic dimensions excitement, depressive and disorganized symptoms′ dimensions. Cannabis use did not associate with duration of untreated psychosis (p=0.443, W= 2709.5). All the analyses were controlled by gender, age and duration of untreated psychosis.

**Discussion:**

Acute and total exposure to cannabis affected deferentially the symptoms dimensions in patients at first episode of psychosis. Previous studies on the relationship between cannabis use and negative symptoms produced mixed results. This may be biased by antipsychotic exposure prior to first assessment. We will investigate the course of the symptoms of those patients to verify if the symptomatic differences are maintained.

